# 肺楔形切除术治疗小体积浸润性肺腺癌患者临床疗效分析

**DOI:** 10.3779/j.issn.1009-3419.2024.102.17

**Published:** 2024-05-20

**Authors:** CUI Shijun, WANG Gaoxiang, HUANG Zhining, WU Mingsheng, WU Hanran, ZHOU Hangcheng, XU Meiqing, XIE Mingran

**Affiliations:** ^1^230001 合肥，安徽医科大学附属省立医院胸外科（崔世军，黄志宁）; ^1^Department of Thoracic Surgery, Anhui Provincial Hospital Affiliated to Anhui Medical University; ^2^230001 合肥，中国科学技术大学附属第一医院胸外科（王高祥，吴明胜，吴汉然，徐美青，解明然）; ^2^Department of Thoracic Surgery, The First Affiliated Hospital of University of Science and Technology of China, Hefei 230001, China; ^3^230001 合肥，中国科学技术大学附属第一医院病理科（周杭城）; ^3^Department of Pathology, The First Affiliated Hospital of University of Science and Technology of China, Hefei 230001, China

**Keywords:** 肺肿瘤, 肺叶切除术, 肺段切除术, 楔形切除术, 疗效, 预后, Lung neoplasms, Lobectomy, Segmentectomy, Wedge resection, Efficacy, Prognosis

## Abstract

**背景与目的:**

随着对肿瘤最大径≤2 cm的非小细胞肺癌的进一步认识和研究，肺段切除术能够达到与肺叶切除术相同的远期预后。但是，针对肺楔形切除术对浸润深度在0.5-1.0 cm的小体积浸润性肺腺癌的预后影响的相关研究较少。因此，本研究主要探讨小体积浸润性肺腺癌患者行楔形切除术的临床疗效与预后。

**方法:**

回顾性分析2016年2月至2017年12月于安徽医科大学附属省立医院胸外科行手术治疗且术后病理结果证实为小体积浸润性肺腺癌的208例患者病历资料。根据手术方式的不同分为肺叶组（n=115）、肺段组（n=48）和楔形组（n=45）三组，采用Kaplan-Meier生存曲线估计法和Cox比例风险回归模型探讨不同手术方式对小体积浸润性肺腺癌术后患者预后的影响。

**结果:**

楔形组与肺段组、肺叶组相比具有更好的围手术期疗效，在术中出血量（P=0.036）、术后引流量（P<0.001）、手术时间（P=0.018）、术后带管时间（P=0.001）、术后并发症发生率（P=0.006）方面差异均有统计学意义。三组患者在生存率（肺叶组 vs 肺段组，P=0.303；肺叶组 vs 楔形组，P=0.742；肺段组 vs 楔形组，P=0.278）和无复发生存率（肺叶组 vs 肺段组，P=0.495；肺叶组 vs 楔形组，P=0.362；肺段组 vs 楔形组，P=0.775）方面无明显差异；单因素及多因素生存分析显示：实性成分占比（consolidation tumor ratio, CTR）是小体积浸润性肺腺癌患者的总生存期和无复发生存期的影响因素（P<0.05）。

**结论:**

对于小体积浸润性肺腺癌患者行楔形切除术可以取得与肺段切除术和肺叶切除术相似的远期预后。当CTR≤0.5时，此类患者优先行楔形切除术。

## Abstract (en)

**Background and objective:**

With further understanding and research into non-small cell lung cancer with tumours ≤2 cm in maximum diameter, segmental lung resection is able to achieve the same long-term prognosis as lobectomy. However, there are few studies on the prognostic effect of wedge resection on small volume invasive lung adenocarcinoma with an invasion depth of 0.5 to 1.0 cm. Therefore, this study focuses on the clinical efficacy and prognosis of wedge resection in patients with small-volume invasive lung adenocarcinoma.

**Methods:**

A retrospective analysis of the medical records of 208 patients who underwent surgery in the Department of Thoracic Surgery of the Affiliated Provincial Hospital of Anhui Medical University from February 2016 to December 2017 was made, and the postoperative pathological results confirmed small volume invasive lung adenocarcinoma. According to their surgical methods, they were divided into lobectomy group (n=115), segmentectomy group (n=48) and wedge resection group (n=45). Kaplan-Meier survival curve estimation and Cox proportional risk regression model were used to explore the influence of different surgical methods on the prognosis of patients with small volume invasive lung adenocarcinoma.

**Results:**

The wedge resection group had better perioperative outcomes compared with the segmentectomy group and lobectomy group, with statistically significant differences in intraoperative bleeding (P=0.036), postoperative drainage (P<0.001), operative time (P=0.018), postoperative time with tubes (P=0.001), and postoperative complication rate (P=0.006). There were no significant differences when comparing the three groups in terms of survival rate (lobectomy group vs segmentectomy group, P=0.303; lobectomy group vs wedge resection group, P=0.742; and segmentectomy group vs wedge resection group, P=0.278) and recurrence-free survival rate (lobectomy group vs segmentectomy group, P=0.495; lobectomy group vs wedge resection group, P=0.362; segmentectomy group vs wedge resection group, P=0.775). Univariate and multivariate survival analyses showed that consolidation tumor ratio (CTR) was the prognostic factor of overall survival and revurrence-free survival for patients with small-volume invasive lung adenocarcinoma (P<0.05).

**Conclusion:**

Wedge resection in patients with small volume invasive lung adenocarcinoma can achieve long-term outcomes similar to segmentectomy and lobectomy. When the CTR≤0.5, wedge resection is preferred in such patients.

据世界卫生组织（World Health Organization, WHO）统计数据^[1,2]^显示，肺癌的全球发病率和死亡率分别位于恶性肿瘤排行榜的第二位和第一位，严重威胁着人们身体健康。其中非小细胞肺癌（non-small cell lung cancer, NSCLC）作为最常见的病理类型，占所有肺癌的80%-85%，而在NSCLC中腺癌占比最多^[3⇓-5]^。对于可切除NSCLC患者，从1995年北美肺癌研究组提出肺癌根治术到目前为止，解剖性肺叶切除术加系统性肺门纵隔淋巴结清扫术仍是治疗该类患者的标准术式^[6,7]^。JCOG0802/WJOG4607L 3期多中心随机对照试验和北美的CALGB140503随机试验的研究^[8,9]^结果表明，对于肿瘤大小<2 cm的NSCLC患者，肺段切除术能够取得与肺叶切除术相似的预后，证实了肺段切除术对该类患者的安全性与有效性。JCOG0804临床试验^[10]^表明，对于肿瘤大小<2 cm、实性成分占比（consolidation tumor ratio, CTR）≤0.25的原位腺癌和微浸润性肺腺癌患者，楔形切除术安全有效，且无需行淋巴结清扫或采样。小体积浸润性肺腺癌^[11,12]^通常指病理程度介于微浸润肺腺癌和浸润性肺腺癌之间、浸润深度在0.5-1.0 cm的腺癌。针对此类患者实行肺楔形切除术是否能达到与肺段切除术及肺叶切除术相似的临床疗效，目前相关研究较少。因此，本研究主要探讨肺楔形切除术治疗小体积浸润性肺腺癌患者的安全性及有效性。

## 1 资料与方法

### 1.1 研究对象

本研究回顾性分析2016年2月至2017年12月于安徽医科大学附属省立医院胸外科行手术治疗且术后病理结果证实为小体积浸润性肺腺癌的523例患者的病历资料。纳入标准：（1）肿瘤大小≤2 cm的周围型肺癌；（2）组织病理学证实为小体积浸润性肺腺癌；（3）行胸腔镜手术；（4）切缘距肿瘤≥2 cm或大于肿瘤的最大直径。排除标准：（1）既往胸部手术史；（2）既往肿瘤病史；（3）开放手术或同时行多部位肺部手术；（4）临床病历资料不完整。

根据纳入和排除标准共纳入208例患者，其中男性88例，女性120例，平均年龄（59.69±0.79）岁。根据手术方式不同分为肺叶组（n=115）、肺段组（n=48）、楔形组（n=45）三组。本研究获安徽医科大学附属省立医院伦理审查委员会批准（伦理号：2023-RE-104），患者知情同意获得豁免。

患者常规术前检查项目包括：心电图、肺功能、肺部计算机断层扫描（computed tomography, CT）平扫+三维重建，必要时行增强CT及骨扫描明确病变性质。CTR值指术前CT影像中肿瘤实性成分最大直径与磨玻璃成分最大直径的比值（CTR测量由2名影像医师分别测量1次，当二者测量结果相差>10%时，由更高年资影像科医师共同参与测量并重新评价后计算得出；对于很不规则的结节，可以用长径和短径的平均值）。

### 1.2 手术方法

本研究纳入患者均行胸腔镜肺部手术。患者采用全身麻醉，健侧卧位，单肺通气。在第4或5肋间腋前线与腋中线之间3 cm切口，置入切口保护套，在单孔下完成手术^[13]^。肺段切除术及肺楔形切除术适用于结节位置位于肺外周1/3带的患者。完整切除肿瘤的切除范围为切缘距肿瘤≥2 cm或大于肿瘤的最大直径。

楔形切除术：自操作孔置入胸腔镜探查，必要时分解胸腔粘连，根据术前影像资料或术前定位确定病灶所在位置，采用切割缝合器在安全切除范围内进行楔形切除。

肺段切除术：自操作孔置入胸腔镜探查，必要时分解胸腔粘连，根据传统方法进行肺段门小结构的辨别，分别对目标肺段静脉、动脉及支气管进行游离显露后，再采用膨肺萎陷法确定段间平面后离断，采用切割缝合器予以靶肺段切除。

肺叶切除术：自操作孔置入胸腔镜探查，必要时分解胸腔粘连，根据传统方法进行肺门结构的辨别，游离暴露出目标肺叶动静脉、支气管以及发育不全叶裂，处理好分支血管、气管后，采用切割缝合器予以靶肺叶切除。

### 1.3 观察指标

分别记录三组患者的一般临床信息，包括年龄、性别、吸烟史、肿瘤位置、肿瘤最大径、术前合并症（糖尿病、脑梗、心律失常等）、CTR、病理亚型、手术方式、淋巴结清扫方式、术中出血量、手术时间、术后引流量、术后带管时间、术后并发症（肺不张、肺部感染、心律失常等）及生存复发情况。浸润深度指瘤体浸润成分最大直径（浸润深度测量由2名病理医师各自测量1次，当二者测量结果相差大于10%时，由更高年资病理科医师共同参与测量确认后，得出结果）；病理亚型采用由国际肺癌研究协会2021年出版的《WHO胸部肿瘤分类》方法，分为贴壁为主型、腺泡或乳头为主型、含有实体或微乳头成分三类。系统性淋巴结清扫常规切除同侧纵隔淋巴结及周围脂肪组织；区域性淋巴结清扫常规清扫术者指定部位的纵隔淋巴结；淋巴结采样摘除视触有明显异常的纵隔淋巴结^[14]^。总生存期（overall survival, OS）指从手术时间开始至因任何原因死亡的时间。无复发生存期（recurrence-free survival, RFS）指从手术时间到肿瘤发生任何方面进展或死亡之间的时间。随访终点为2022年12月31日。

### 1.4 统计学方法

采用SPSS 26.0统计学软件进行数据分析。符合正态分布资料以Mean±SD表示，计量资料比较采用t检验。偏态分布资料采用中位数（P25, P75）进行统计描述，组间比较采用Wilcoxon秩和检验进行。计数资料比较采用卡方检验。采用Kaplan-Meier法绘制生存曲线并计算生存率，组间生存率差异以Log-rank检验分析并作趋势检验。采用Cox模型进行多因素生存分析。P<0.05为差异有统计学意义。

## 2 结果

### 2.1 一般临床资料对比

三组患者在年龄、性别、吸烟史、肿瘤位置、肿瘤最大径、术前合并症、CTR及病理亚型等方面差异均无统计学意义（P>0.05）。全组未见淋巴结转移。肺叶组行系统性清扫64例，有限性淋巴结清扫51例；肺段组行系统性清扫14例，有限性淋巴结清扫28例，不清扫6例；楔形组均未行清扫淋巴结，差异有统计学意义（P<0.001），见表1。

**表 1:** 小体积浸润性肺腺癌患者一般临床资料对比

Factors	Lobectomy (n=115)	Segmentectomy (n=48)	Wedge resection (n=45)	X^2^	P
Age (yr)				0.312	0.855
≤65	74 (64.35%)	33 (68.75%)	29 (64.44%)		
>65	41 (35.65%)	15 (31.25%)	16 (35.56%)		
Gender				0.342	0.843
Male	47 (40.87%)	22 (45.83%)	19 (42.22%)		
Female	68 (59.13%)	26 (54.17%)	26 (57.78%)		
Smoking				3.907	0.142
Yes	14 (12.17%)	4 (8.33%)	1 (2.22%)		
No	101 (87.83%)	44 (91.67%)	44 (97.78%)		
Tumor location				8.639	0.374
Right upper lung	46 (64.35%)	16 (33.33%)	17 (37.78%)		
Right middle lung	14 (12.17%)	2 (4.17%)	6 (13.33%)		
Right lower lung	14 (12.17%)	9 (18.75%)	2 (4.44%)		
Left upper lung	25 (21.74%)	15 (31.25%)	14 (31.11%)		
Left lower lung	16 (13.91%)	6 (12.50%)	6 (13.33%)		
Tumor size (cm)				1.509	0.470
Diameter≤1	44 (38.26%)	20 (41.67%)	22 (48.89%)		
1<Diameter≤2	71 (61.74%)	28 (58.33%)	23 (51.11%)		
Preoperative comorbidities				0.784	0.676
Yes	43 (37.39%)	16 (33.33%)	19 (42.22%)		
No	72 (62.61%)	32 (66.67%)	26 (57.78%)		
CTR				2.115	0.622
CTR≤0.5	79 (68.70%)	26 (54.17%)	32 (71.11%)		
0.5<CTR<1.0	36 (31.30%)	22 (45.83%)	13 (28.89%)		
Pathologic subtype				1.141	0.565
Lepidic	68 (59.13%)	26 (54.17%)	27 (60.00%)		
Acinar or papillary	41 (35.65%)	20 (41.67%)	18 (40.00%)		
Solid or micropapillary	6 (5.22%)	2 (4.17%)	0 (0.00%)		
Lymphadenectomy				18.246	<0.001
Systematic cleaning	64 (55.65%)	14 (29.17%)	0 (0.00%)		
Limited cleaning	51 (44.35%)	28 (58.33%)	0 (0.00%)		
No cleaning	0 (0.00%)	6 (12.50%)	45 (100.00%)		

CTR: consolidation tumor ratio.

### 2.2 围手术期资料比较

楔形组与肺段组、肺叶组相比具有更少的术中出血量和术后引流量、更短的手术时间和术后带管时间以及更低的术后并发症发生率，差异均有统计学意义（P值分别为0.036、<0.001、0.018、0.001、0.006，见表2）。

**表 2:** 小体积浸润性肺腺癌患者围手术期资料对比

Factors	Lobectomy (n=115)	Segmentectomy (n=48)	Wedge resection (n=45)	X^2^/Z	P
Intraoperative haemorrhage (mL)	51.6 (20.3, 79.8)	50.3 (19.2, 96.8)	29.8 (13.2, 47.6)	0.175	0.036
Operating time (min)	155.1 (147.7, 180.2)	138.7 (109.7, 206.3)	49.2 (26.0, 68.4)	0.224	0.018
Postoperative drainage (mL)	652.6 (485.7, 896.3)	492.4 (414.6, 731.2)	188.4 (165.3, 312.8)	2.639	<0.001
Postoperative time with tube (d)	4.6 (3.1, 7.5)	2.9 (2.6, 7.3）	2.1 (1.6, 3.4)	3.622	0.001
Postoperative complications				4.628	0.006
Yes	22 (19.13%)	7 (14.58%)	3 (6.67%)		
No	93 (80.87%)	41 (85.42%)	42 (93.33%)		

### 2.3 复发及生存预后分析

随访期间肺叶组、肺段组和楔形组分别有13、5和4例患者出现局部复发。随访时间范围为60.0-82.0个月，中位随访时间为64.0个月。全组患者第1、3和5年生存率分别为100.0%、100.0%和94.7%；肺叶组第1、3和5年生存率分别为100.0%、100.0%和93.9%，肺段组第1、3和5年生存率分别为100.0%、100.0%和93.8%，楔形组第1、3和5年生存率分别为100.0%、100.0%和97.8%；Log-rank法两两比较组间生存率差异均无统计学意义（肺叶组 vs 肺段组，P=0.303；肺叶组 vs 楔形组，P=0.742；肺段组 vs 楔形组，P=0.278），见图1A。

**图 1:**
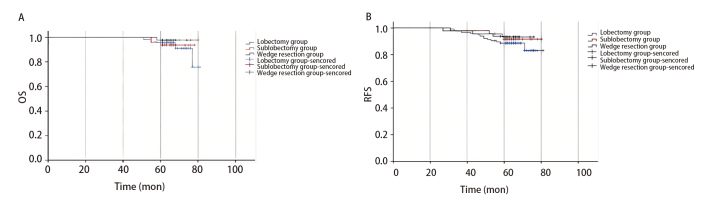
不同手术方式小体积浸润性肺腺癌患者生存曲线。A：OS生存曲线；B：RFS生存曲线。

全组患者第1、3和5年RFS率分别为100.0%、97.6%和90.4%；肺叶组第1、3和5年RFS率分别为100.0%、97.4%和90.4%，肺段组第1、3和5年RFS率分别为100.0%、97.9%和93.8%，楔形组第1、3和5年RFS率分别为100.0%、97.8%和93.3%；Log-rank法两两比较组间RFS率差异均无统计学意义（肺叶组 vs 肺段组，P=0.495；肺叶组 vs 楔形组，P=0.362；肺段组 vs 楔形组，P=0.775），见图1B。

### 2.4 影响小体积浸润性肺腺癌患者预后的单因素和多因素分析

将小体积浸润性肺腺癌患者基线资料进行单因素分析发现，CTR是小体积浸润性肺腺癌患者OS与RFS的影响因素（P=0.012, P=0.025）；其余因素均不是小体积浸润性肺腺癌患者生存率影响因素（P>0.05），见表3。通过Cox模型将CTR、病理亚型、手术方式、淋巴结清扫方式进行多因素分析后发现，CTR是小体积浸润性肺腺癌患者OS（HR=0.197, 95%CI: 0.052-0.741, P=0.016）与RFS（HR=0.315, 95%CI: 0.126-0.788, P=0.014）的影响因素。

**表 3:** 小体积浸润性肺腺癌患者OS、RFS单因素分析

Factors	n	OS [Mean (95%CI), mon]	P	RFS [Mean (95%CI), mon]	P
Age (yr)			0.097		0.957
≤65	136	79.6 (78.3-80.9)		78.3 (76.7-80.0)	
>65	72	76.3 (73.1-79.6)		72.1 (68.5-75.7)	
Gender			0.272		0.199
Male	88	78.8 (77.2-80.4)		78.0 (76.3-79.6)	
Female	120	78.7 (77.3-80.2)		75.8 (73.2-78.5)	
Smoking			0.415		0.844
Yes	19	77.3 (74.3-80.3)		78.5 (75.6-81.4)	
No	189	79.4 (78.3-80.5)		77.0 (75.1-78.8)	
Tumor location			0.613		0.971
Right upper lung	79	78.2 (76.6-79.8)		72.2 (68.7-75.8)	
Right middle lung	22	73.3 (71.1-75.5)		74.0 (72.2-75.9)	0.879
Right lower lung	25	80.0 (78.1-81.9)		76.0 (71.2-80.8)	0.544
Left upper lung	54	76.3 (74.5-78.2)		75.5 (73.9-77.2)	0.897
Left lower lung	28	76.0 (74.2-77.9)		73.7 (69.2-78.1)	0.931
Tumor size (cm)			0.298		0.235
Diameter≤1	86	80.2 (79.2-81.1)		78.2 (75.9-80.5)	
1<Diameter≤2	122	77.4 (75.6-79.2)		75.7 (73.6-77.9)	0.221
Preoperative comorbidities			0.599		0.171
Yes	78	78.6 (75.2-81.9)		77.9 (75.7-80.0)	
No	130	78.1 (76.7-79.4)		76.0 (74.0-78.1)	
CTR			0.012		0.025
CTR≤0.5	137	78.9 (75.3-80.7)		76.5 (75.3-80.7)	
0.5<CTR<1.0	71	76.0 (71.5-79.5)		76.1 (73.5-79.5)	
Pathologic subtype			0.074		0.357
Lepidic	121	80.2 (78.1-81.6)		77.2 (75.1-80.6)	
Acinar or papillary	79	78.3 (74.2-80.8)		76.8 (72.2-78.8)	
Solid or micropapillary	8	62.7 (59.4-65.3)		72.6 (69.4-73.5)	
Lymphadenectomy			0.989		0.301
Systematic cleaning	78	77.6 (74.1-81.2)		76.3 (72.3-80.3)	
Limited cleaning	79	78.6 (77.6-79.6)		76.5 (74.7-78.2)	
No cleaning	51	76.8 (74.2-78.5)		76.2 (73.6-79.7)	
Operative approach			0.977		0.811
Lobectomy	115	76.3 (72.3-80.3)		75.3 (72.8-78.6)	
Segmentectomy	48	76.5 (74.7-78.2)		78.6 (76.3-80.9)	
Wedge resection	45	76.2 (73.6-79.7)		77.9 (75.62-80.2)	

CI: confidence interval.

## 3 讨论

肺叶切除术加系统性肺门纵隔淋巴结清扫术一直是可切除肺腺癌患者的主要治疗方式。近年来，随着低剂量胸部CT用于早期肺癌的筛查，更多的IA期NSCLC患者被检出^[15]^，对影像学预测的恶性程度较低的早期肺癌可以进行局限性切除，使得肺叶切除术的地位受到了质疑。因此，针对其标准术式的探讨一直是业内热点问题之一。越来越多的研究^[16⇓-18]^显示，对于≤2 cm的NSCLC患者行亚肺叶切除术及肺叶切除术能够取得相似的远期预后，亚肺叶切除术可作为≤2 cm的NSCLC患者的标准术式。在亚肺叶切除术中，肺楔形切除术是肺癌治疗的重要环节与手段，其手术操作更方便、流程更简化、切除肺组织更少，具有更小的手术创伤和肺功能损害以及更好的术后生活质量等明显优势^[19⇓-21]^。JCOG0802/WJOG4607L 3期多中心随机对照试验和CALGB140503随机试验^[8,9]^证实亚肺叶切除术对于肿瘤大小<2 cm的浸润性肺腺癌安全有效；而JCOG0804试验^[10]^证实楔形切除术对于原位腺癌、微浸润性肺腺癌患者安全有效。本研究发现，对于病理程度介于微浸润肺腺癌和浸润性肺腺癌之间的小体积浸润性肺腺癌患者行楔形切除术可以取得与肺段切除术、肺叶切除术相比更好的近期疗效和相似的远期预后。

本研究发现，楔形切除术相比于肺段切除术和肺叶切除术在小体积浸润性肺腺癌患者中所需手术时间和术后带管时间更短，术后并发症发生率更低，而这三种术式在OS和RFS方面无显著差异，这意味着楔形切除术可以在取得比肺段切除术、肺叶切除术更好的围手术期效益的同时，还具有不劣于肺段切除术和肺叶切除术的远期预后的优势，对于此类患者可优先行楔形切除术。Shi等^[22]^的一项手术切除治疗IA期NSCLC的荟萃分析认为当肿瘤直径≤2 cm时，虽然不能证明楔形切除术相比于肺段切除术、肺叶切除术所取得的远期预后更好，但三者预后无明显差异。Deng等^[23]^也认为对于肿瘤直径<2 cm的IA期NSCLC患者行楔形切除术的术后OS和RFS与肺段切除术、肺叶切除术预后相似，相对来说，楔形切除术在手术时间、术后并发症及肺功能保护方面具备优势，该类患者可优先行楔形切除术。

CTR值是目前肺恶性结节重要评估指标，CTR值的不同往往预示着预后、复发、手术方式的不同。本研究通过单因素和多因素分析发现，CTR是小体积浸润性肺腺癌患者的OS和RFS的影响因素。当CTR≤0.5时，小体积浸润性肺腺癌患者的OS和RFS均达到98%，可以获得更好预后，这与JCOG0802/WJOG4607L 3期多中心随机对照试验^[12]^的结果保持一致。Bian等^[24]^也在一项基于单中心楔形切除术治疗外周IA期肺腺癌的疗效和安全性的真实世界研究中表明，CTR是其独立预后因子，受试者工作特征（receiver operating characteristic, ROC）曲线所得出CTR的临界值为0.6，当CTR<0.6时，该类患者取得的预后更好。

本研究发现病理亚型不是小体积浸润性肺腺癌患者OS和RFS的影响因素。在本研究中，小体积浸润性腺癌患者所有病理亚型的整体预后较好，其中以贴壁型为主型患者数量最多、预后最好；腺泡型为主型或乳头型为主型预后较好，含微乳头型和实体型为主型预后最差；既往研究^[12,20]^对于I期浸润性肺腺癌病理亚型的分析也得出了相同的结果，与本研究不一致的是他们认为病理亚型是I期浸润性肺腺癌的预后危险因素，其中微乳头成分≥5%是影响肿瘤复发和生存的重要危险因素，这可能和本研究只纳入了IA期肺腺癌患者，且微乳头型为主型和实体型为主型仅占8例，不能完全反映肺腺癌患者远期预后情况有关。

本研究认为以上结果的原因可能在于：（1）对于小体积浸润性肺腺癌，病理程度介于微浸润肺腺癌和浸润性肺腺癌之间，整体病理分期较早且病理亚型多为以贴壁与腺泡生长亚型为主，该类病例含微乳头成分≥5%、纯实性等预后高危因素少，整体预后均较好。（2）手术过程中保证足够的切缘，在组织病理学上确保肿瘤被完全切除，所以行楔形切除术可以取得与肺段切除术、肺叶切除术相似的预后^[25,26]^。（3）术中冰冻结果可以进行术式的优化与选择，更加科学地确定切除范围也能帮助该类患者取得更好的预后。对于IA期小体积肺腺癌患者在保证切缘足够且CTR≤0.5的前提下，楔形切除术可以达到与肺段切除术和肺叶切除术相似的远期预后^[27,28]^。对于术中病理结果提示为小体积浸润性腺癌，但CTR≥0.5时，可根据术中实际情况（如：含有微乳头成分、纯实性或肿大淋巴结）来决定是否要进行扩大切除。

本研究发现淋巴结清扫方式对小体积浸润性肺腺癌患者OS和RFS没有明显影响。Li等^[29]^在对肺原位腺癌或微浸润肺腺癌患者术后进行长达10年随访后认为楔形切除术对其是可治愈的，无论是进行淋巴结清扫还是采样，二者所取得预后是相似的。Wang等^[30]^研究发现对直径≤1 cm的cI期NSCLC患者淋巴结转移率低，术中可不进行淋巴结清扫。日本JCOG1211研究结果^[20]^表示对于纯磨玻璃结节为主型的肺癌，当CTR值≤0.5，可不对纵隔淋巴结进行清扫或采样。针对小体积浸润性肺腺癌患者淋巴结转移发生率较低，行肺楔形切除术可不行淋巴结清扫或采样。但是，针对术前有影像学检查及术中冰冻等提示存在高危因素的患者，建议行选择性淋巴结清扫^[31]^。

本研究为单中心小样本回顾性研究，存在一定的数据有限与选择偏差的局限性，需要多中心前瞻性随机试验来验证。此外，本研究定义瘤体浸润性成分的大小是小体积浸润性肺腺癌定义权重最高的变量，对于此类患者预后参数究竟是肿瘤组织的病理形态变化更重要还是肿瘤的大小更重要，可能还需要更多的真实世界研究证据来揭示。

综上所述，对于小体积浸润性肺腺癌患者行楔形切除术可以取得与肺段切除术和肺叶切除术相似的远期预后。因此，当CTR≤0.5时，小体积浸润性周围型肺腺癌患者优先行楔形切除术。
